# Pediatric sleep disturbances and treatment with melatonin

**DOI:** 10.1186/s12967-019-1835-1

**Published:** 2019-03-12

**Authors:** Susanna Esposito, Daniela Laino, Renato D’Alonzo, Annalisa Mencarelli, Lorenza Di Genova, Antonella Fattorusso, Alberto Argentiero, Elisabetta Mencaroni

**Affiliations:** 1 Pediatric Clinic, Department of Surgical and Biomedical Sciences, Università degli Studi di Perugia, Piazza Menghini 1, 06129 Perugia, Italy; 2 Department of Pathophysiology and Transplantation, Università degli Studi di Milano, Milan, Italy

**Keywords:** Attention-deficit/hyperactivity disorder, Autism spectrum disorders, Insomnia, Melatonin, Neurodevelopmental disabilities, Sleep disturbances

## Abstract

**Background:**

There are no guidelines concerning the best approach to improving sleep, but it has been shown that it can benefit the affected children and their entire families. The aim of this review is to analyse the efficacy and safety of melatonin in treating pediatric insomnia and sleep disturbances.

**Main body:**

Sleep disturbances are highly prevalent in children and, without appropriate treatment, can become chronic and last for many years; however, distinguishing sleep disturbances from normal age-related changes can be a challenge for physicians and may delay treatment. Some published studies have shown that melatonin can be safe and effective not only in the case of primary sleep disorders, but also for sleep disorders associated with various neurological conditions. However, there is still uncertainty concerning dosing regimens and a lack of other data. The dose of melatonin should therefore be individualised on the basis of multiple factors, including the severity and type of sleep problem and the associated neurological pathology.

**Conclusions:**

Melatonin can be safe and effective in treating both primary sleep disorders and the sleep disorders associated with various neurological conditions. However, there is a need for further studies aimed at identifying the sleep disordered infants and children who will benefit most from melatonin treatment, and determining appropriate doses based on the severity and type of disorder.

## Background

Sleep disturbances are highly prevalent in children and, without proper treatment, can become chronic and last for many years [1]; however, distinguishing sleep disturbances from normal age-related changes can be a challenge for physicians and may delay treatment. The definition of insomnia is repeated difficulty in getting to sleep (more than 30 min per night), insufficient sleep (less than 8 h), and poor sleep consolidation or quality despite having the age-appropriate time and opportunity to sleep, all of which lead to daytime functional impairment for both patients and their families [2]. Sleepiness in children may manifest as irritability, behavioural problems, learning difficulties, motor vehicle crashes (teenagers), and poor academic performance [3].

Sleep behaviour has some general trends, and the large variations among children may be due to chronic anxiety and cultural and/or genetic differences. Epidemiological studies indicate that the prevalence of pediatric insomnia varies from 10% in Vietnam and Thailand to 25–30% in United States and Australia, and as much as 75% in China and Taiwan [4–6]. Knowing the normal developmental stages of sleep is therefore useful in distinguishing normal sleep from frequent sleep disturbances. Table 1 shows the normal parameters of sleep in children and adolescents.

**Table 1:** Normal sleep parameters in children and adolescents

Age	Total sleep time	Naps (on average)
0–2 months	16–18 h	3.5 per day at 1 month of age
2–12 months	12–16 h Most children aged 6–9 months sleep throughout the night	Two per day at 12 months of age
1–3 years	10–16 h	One per day at 18 months of age
3–5 years	11–15 h	50% of 3-year-olds do not nap
5–14 years	9–13 h	5% of whites and 39% of blacks nap at 8 years of age
14–18 years	7–10 h	Napping at this age suggests insufficient sleep or a possible sleep disorder

There are no guidelines concerning the best approach to improving sleep, but it has been shown that it can benefit the affected children and their entire families. The aim of this review is to analyse the efficacy and safety of melatonin in treating pediatric insomnia and sleep disturbances.

## Pediatric sleep disturbances

The most frequent cause of pediatric insomnia is behavioural insomnia of childhood (BIC), which affects both getting to sleep and maintaining sleep [7]. The type of BIC associated with sleep onset is characterised by a child’s dependence on specific stimuli, objects, or settings in order to fall asleep or return to sleep after awakening. The factors involved in a child’s inability to fall asleep on his/her own include a need for parental presence while falling asleep, intentional co-sleeping, or feeding at the time of sleep onset [8]. The limit-setting type of BIC is characterised by bedtime stalling or refusal due to inadequate limit setting by a caregiver, prolonged sleep onset, and occasional overnight awakenings, and typically occurs in children of pre-school or school age. The mixed types of BIC are characterised by features of both of the other types.

Psycho-physiological insomnia is characterised by heightened arousal and learned sleep prevention [8]. Affected children may sleep more easily in settings other than their bedrooms or have an easier time falling asleep when they are not trying to do so. They may also worry about their sleep and show excessive cognitive and somatic arousal.

Other sleep disorders are due to circadian rhythm abnormalities [9]. One example is delayed sleep–wake phase disorder, which is particularly observed in adolescents and is characterised by a tendency to go to bed late and difficulties in waking up; such children may also find it difficult to remain alert during the morning. Circadian disorders are also frequently seen in children with developmental disabilities.

### Biological causes of pediatric sleep disorders

Hyper-arousal and hypersensitivity can contribute to insomnia, particularly in children with autism spectrum disorders (ASDs) and multiple dysregulated neurotransmitters, such as gamma-aminobutyric acid (GABA), melatonin and serotonin [10]. The prevalence of insomnia among these children is 50–80%, which is also partially due to the role played by chronic anxiety [11, 12]. Affected children may be hypersensitive to environmental stimuli, hyper-arousal and/or have difficulties with self-regulation.

### Medical causes of pediatric sleep disorders

Insomnia and sleep disorders may also be caused by gastrointestinal and pain-related diseases, pulmonary diseases such as asthma or chronic cough, and upper airway pathologies, especially snoring and obstructive sleep apnea (OSA). Recent studies have found that disturbed sleep is frequent in children with atopic dermatitis (AD), a major factor that impairs their quality of life and can have negative effects on neurocognitive function and behaviour. This may not only be due to pruritus and scratching, but may also involve the circadian rhythm of cytokines, the immune system, skin physiology such as transcutaneous water loss and skin blood flow, and even environmental factors [13].

There is a clear association between insomnia and hypertension, although the rate of nocturnal hypertension is significantly higher in older than younger patients (63.1% vs 41.1%) [14].

Children with Down syndrome have an increased prevalence of sleep disorders, including insomnia, and experience difficulty in getting to sleep, troublesome awakenings in the night, and early morning awakenings [15]. A number of neurological diseases can influence sleep, including headaches, epilepsy and restless legs syndrome [16], and psychiatric disorders such as anxiety, depression, and post-traumatic disorders can also alter a child’s sleeping ability; conversely, insomnia can worsen these disturbances [16].

Furthermore, up to 80% of children with neurodevelopmental disorders (NDDs), including ASDs, cerebral palsy, Rett syndrome, Angelman syndrome, Williams syndrome, and Smith–Magenis syndrome (SMS) are affected by disrupted sleep, which frequently has deleterious effects on their daytime behaviour, cognition, growth, and overall development. The etiology of sleep disorders in children with NDDs is highly heterogeneous and disease specific [17].

Finally, some drugs can interfere with sleep onset and maintenance [18].

### Behavioural causes of pediatric sleep disorders

The behavioural causes of insomnia include the use of electronic devices, caffeine and alcohol, irregular bedtimes, and a lack of physical exercise [6], all of which may overlap with biological causes: e.g. a dependence on external factors to fall and stay asleep (anxiety requiring the presence of caregivers).

Table 2 shows the characteristics of the principal pediatric sleep disorders.

**Table 2:** Common sleep disorders in children

Sleep disorder	Epidemiology	Clinical features	Diagnostic criteria	Treatment options
Obstructive sleep apnea	Prevalence: 1–5% Onset: 2–8 years of age M:F = 1:1 More frequent in blacks and people with craniofacial abnormalities, Down syndrome, neuromuscular diseases, or choanal atresia	Snoring Unusual sleep positions Sleep-related paradoxical breathing Bedtime enuresis or diaphoresis Morning headaches Cognitive/behavioural problems Excessive daytime sleepiness Enlarged adenoids and tonsils Pectus excavatum	PSG apnea–hypopnea index > 1.5 per hour	Adeno-tonsillecto-my CPAP, nasal steroids, rapid maxillary expansion
Confusional arousals	Prevalence: 17.3% in 3–13-year-olds, 2.9–4.2% in children older than 15 years M:F = 1:1 Positive family history	Sleep drunkenness Unusual behaviour Slowed responsiveness Slurred speech Confused after awakening Occurs during the first half of the sleep period, no memory of the event	History	Re-assurance Increase total sleep time Scheduled awakenings Bedroom/home safety counselling
Sleep terrors	Prevalence: 1–.5% Onset: early childhood M:F = 1:1	Intense fear Difficulty in awakening from episode Dangerous activities Occurs during the first half of the sleep period, no memory of the event Overlap with other parasomnias	History	Re-assurance Increase total sleep time Scheduled awakenings Bedroom/home safety counselling -Benzo-diazepines
Nightmares	Prevalence: 10–50% in 3–5-year-olds Onset: 3–6 years of age; peaks: 6–10 years of age M:F = 1:1	Unpleasant dreams Increased sympathetic activity Occurs during the second half of the sleep period, memory of the event Reluctance to sleep increases Association with mood disorders or post-traumatic stress disorder	History	Re-assurance Increase total sleep time Scheduled awakenings Bedroom/home safety counselling Cognitive behavioural therapy, SSRI (off-label use)
Behavioural insomnia of childhood	Prevalence: 10–30% M:F = 1:1	Sleep-onset association type Limit-setting type	History	Prevention, parental education, and extinction techniques
Delayed sleep phase disorder	Prevalence: 7–16% in adolescents Onset: adolescence Positive familiar history in 40% of cases	Difficulty in falling asleep and waking up at socially acceptable times Night owl	History Sleep diary and/or actigraphy for at least 1 week	Sleep hygiene education Regular sleep–wake schedule Avoid bright lights before bedtime Melatonin Bright light therapy Use of sleep logs to monitor progress
Restless legs syndrome	Prevalence: 2% More common in F Positive familiar history	Urge to move legs with discomfort Begins in the evening, worsens with rest, eases with movement Association with iron deficiency Association with negative behaviour and mood, and decreased cognition and attention Increased prevalence in children with ADHD	History PSG Presence of two of the following: disturbed sleep; a first-degree relative with the condition; five or more periodic limb movements per hour of sleep during PSG	Avoid nicotine and caffeine Dis-continue offending medications Iron replacement in the case of deficiency Severe cases: levodopa, dopamine agonists, gabapentin

*PSG* polysomnography, *CPAP* continuous positive airway pressure, *M* males, *F* females, *SSRI* selective serotonin re-uptake inhibitors, *ADHD* attention deficit/hyperactivity disorder

## Melatonin treatment for pediatric sleep disorders

### Physiology of melatonin

Melatonin is an indole hormone that is enzymatically synthesised from the amino acid tryptophan in the pineal gland [19]. Its secretion is regulated by the supra-chiasmatic nucleus (SCN) in the hypothalamus, the site of the biological clock. Melatonin is often referred to as “the hormone of darkness” because its synthesis and secretion is enhanced by darkness and inhibited by light. The timing of melatonin production is influenced by the retinal perception of light and the endogenous rhythmicity of neurons within the SCN, which controls the pineal gland via neural signals in a multi-synaptic pathway. This control system allows the duration and timing of melatonin secretion to change with the seasons: its duration is longer during the short days of winter than during the long days of summer [20]. Even domestic lighting can inhibit its production and secretion [21].

Melatonin plays a key role in regulating circadian rhythm, and is involved in other biological functions as a result of its chronobiotic and anti-oxidant properties, anti-inflammatory effects, and free radical scavenging. It is critically involved in early development. and regulates the vigilance states that depend on the activated melatonin receptors (MT1 and MT2) [22]. A study by Sadeh et al. has demonstrated that infants with an immature pattern of melatonin secretion show a delayed peak in melatonin levels that is associated with more fragmented sleep during the night, thus suggesting that melatonin plays a major role in the development of sleep–wake rhythm [23]. LeBourgeois et al. has shown that toddlers with a later dim-light melatonin onset (DLMO) had later bedtimes, sleep onset times, mid-sleep times, and wake times [24].

Melatonin concentrations are very low during the first 3 months of life and then abruptly increase, probably because the melatonin in human milk has a clear circadian curve and contributes to consolidating the sleep–wake rhythm of infants until their own circadian system matures [25]. Endogenous nocturnal melatonin levels are considerably lower in adults than in children [26], which is due more to their greater body size than a reduction in pineal secretion.

### Pharmacokinetics and mechanism of action of melatonin

Pharmacokinetics and mechanism of action of melatonin have been studied in adults. The intravenous administration of melatonin allows its rapid distribution (half-life approximately 0.5–5.6 min) and elimination, whereas oral administration leads to peak plasma concentrations within 60 min [27]. Within 1 h of the ingestion of 1–5 mg, melatonin concentrations are 10–100 times higher than their physiological nocturnal peak, and return to basal levels 4–8 h. Melatonin is rapidly metabolised, mainly in the liver by microsomal enzyme P450 (which may explain its potential interactions with anti-epileptic drugs) and secondarily in the kidney. It undergoes hydroxylation to 6-hydroxymelatonin followed by conjugation with sulfuric acid (90%) or glucuronic acid (10%), and is excreted in urine (approximately 5% of serum melatonin is excreted unchanged). Its principal metabolite 6-sulfatoxymelatonin (6-SM) is inactive, and its urinary excretion reflects plasma melatonin concentrations [28].

Melatonin’s most characterised mechanism of action is the activation of two membrane-specific receptors: high-affinity ML1 and low-affinity ML2 [29]. The ML1 receptor has two sub-types: Mel1a (or MT1) and Mel1b (or MT2). The first is widely distributed in the *pars tuberalis* of the anterior pituitary, the SCN of the hypothalamus, the cortex, the thalamus, substantia nigra, nucleus accumbens, amygdala, hippocampus, cerebellum, cornea and retina; the second is mainly distributed in the retina and secondarily in the hippocampus, cortex, paraventricular nucleus, and cerebellum [30]. Melatonin receptors have also been detected in peripheral tissues, including the heart and arteries, adrenal gland, kidney, lung, liver, gallbladder, small intestine, adipocytes, ovaries, uterus, breast, prostate, and skin [31], as well as in T and B cells. There is considerable between-species variation in the density and location of melatonin receptors [29], and the effects of melatonin depend on the localisation and type of receptor.

## Melatonin in neuropsychiatric disorders

### Attention-deficit/hyperactivity disorder (ADHD)

Up to 70% of children with ADHD have mild to severe sleep problems. A meta-analysis of sleep disorders in children and adolescents with ADHD found that they had significantly more problems than those without [32], with it has been shown that children with ADHD have increased levels of daytime sleepiness and that sleep onset insomnia is arguably the most frequent cause [33].

The reasons for sleep onset insomnia in children with ADHD are heterogeneous and multifactorial [34]. There is currently no consensus as to how to treat sleep disorders in ADHD, and only low-grade empirical evidence is available. Melatonin may be an option if the insomnia is related to a delayed sleep phase disorder [35]. Studies have shown that treatment with 3–6 mg of melatonin per night significantly reduces sleep onset delay and increases total sleep duration [36].

Melatonin seems to be predominant in hyperactive-impulsive/conduct disordered children (PHI/CD) of the ADHD subtype without the influence of co-morbid depressive symptoms. Methylphenidate can increase sleep problems in children with ADHD because insomnia is the main adverse effect of this drug [37]. Punja et al. have published the results of a randomised controlled trial that provide rigorous evidence of the effectiveness of melatonin in children with ADHD who experience initial insomnia [38].

### Autism spectrum disorders and associated genetic conditions (ASD)

Sleep disorders are frequent in ASD patients, with prevalence estimates ranging from 30% to 53% [39, 40], and can worsen the symptoms of autism. Abnormalities in daytime or night-time melatonin levels in comparison with typically developing controls have often been reported, which suggests that melatonin supplementation may improve sleep parameters in children with ASDs.

Gene abnormalities that could contribute to decreasing melatonin production have been reported in a subgroup of children with ASDs and concomitant sleep onset delay [41]. One study of girls with Rett syndrome found that treatment with melatonin 2.5–7.5 mg decreased sleep onset time and total sleep duration [42], but studies of children with tuberous sclerosis (a genetic condition associated with an increased risk of ASD) have found that melatonin treatment does not decrease sleep onset latency, although it does increase the duration of night-time [43, 44]. These studies suggest that the etiology of ASD and a deficiency in acetyl-serotonin-N-methyl-transferase (the enzyme that catalyses the final reaction in melatonin biosynthesis) may affect the efficacy of melatonin in some autistic subjects, and that (when known) etiology should be considered when considering it as a treatment option.

The majority of children with ASDs respond to a dose of 1–3 mg administered 30 min before bedtime, which improves sleep onset latency and total sleep duration in about 80% of cases. Melatonin is well tolerated and has minimal adverse effects [45]. Cortesi et al. studied 134 children (69 of whom received controlled-release melatonin 3 mg) in the largest randomised and placebo-controlled trial conducted so far, and found that the treatment reduced sleep onset times and increased sleep duration and efficiency [46]. These improvements were observed in children who received cognitive behavioural therapy and/or melatonin, but not in the children receiving placebo.

A recent meta-synthesis of studies of the efficacy of sleep-based interventions in children with ASDs concluded that melatonin, behavioural interventions and parent education/interventions are the most effective means of improving multiple sleep domains [47]. Using melatonin to treat ASD patients with sleep disorders is particularly interesting because of the reported abnormalities in central and peripheral serotonin neurobiology, although the association between melatonin and serotonin needs to be clarified. Further research into the sleeping problems of ASD patients is required in order to elucidate the mechanism of action of melatonin supplementation and identify the patients who are most likely to benefit from melatonin treatment [48].

### Melatonin in patients with neurodevelopmental disabilities (NDDs)

The sleep disorders observed in 13–86% of patients with NDDs [49] are often complex and usually more difficult to treat than in subjects without NDDs. Melatonin is widely used in children with insomnia and NDD because of its apparent safety, but there are no specific clinical guidelines as to how to prescribe it to such patients [50, 51].

Approximately half of the studies of children with NDDs stemming from various disorders/syndromes have reported that melatonin treatment increases sleep duration, but the other half found no difference in comparison with placebo. Two studies found that the treatment reduced night awakenings, but three did not find any significant reduction [52–56].

Altered endogenous melatonin profiles have been reported in patients with Down syndrome, Prader-Willi syndrome, and Sanfilippo syndrome [57], but there is very little information concerning the efficacy or safety of melatonin treatment in children with these conditions. There is therefore a need for further trials aimed at evaluating the efficacy of melatonin in such children and determining whether there are any between-syndrome differences in melatonin metabolism.

Previous studies of circadian rhythm problems in children with SMS have reported higher endogenous melatonin levels during the day than during the night [58]. Evidence that melatonin treatment combined with a beta1-adrenergic antagonist may help children with SMS and inverted or altered circadian patterns has been provided by a study examining the open-label use of prolonged-release melatonin in 88 children with neurodevelopmental disorders, 47 of whom were affected by SMS [59]. The results revealed improvements in parent-reported sleep latency, sleep duration, night awakenings, and sleep quality.

There are two small studies of children with Angelman syndrome: the first was an open-label trial involving 13 children in whom sleep duration increased during treatment with melatonin 3 mg [60]; the second reported that treatment melatonin 2.5–5 mg led to shorter sleep onset latencies, earlier sleep onset times, fewer night awakenings, and longer sleep duration in comparison with placebo [61]. However, once again, more research is required before drawing any conclusions about the efficacy and safety of melatonin treatment in children with Angelman syndrome.

Disturbed sleep is frequent in patients with Williams syndrome, but its exact etiology is unknown. A number of studies have found that children with Williams syndrome have more profound sleep disturbances than typically developing children, including increased sleep anxiety, bedtime resistance, sleep onset delay, frequent night-time awakenings and night walking, repetitive leg movement, restlessness and arousal [62, 63]. However, the efficacy of melatonin in this group of children has not yet been described.

### Melatonin in patients with epilepsy

There is great interest in determining whether melatonin reduces the exacerbation of seizures. A double-blind, placebo-controlled crossover trial investigating the effect of melatonin on seizures in 12 patients with uncontrolled epilepsy observed a significant reduction in diurnal seizures [64]; however, subsequent reviews have concluded that it is was not possible to draw any definite conclusions concerning the role of melatonin in reducing seizure frequency or improving the quality of life of people with epilepsy [65–67].

A reduction in intrinsic melatonin levels has been reported in patients with epilepsy, which may explain why melatonin supplementation improves sleep in such patients [68]. It has also has been suggested that melatonin has GABA agonist effects that may inhibit arousal systems during sleep [69]. On the basis of these findings, Jain et al. carried out a randomised, double-blind cross-over study of 11 children with epilepsy and sleep disturbances [70], the results of which indicated that melatonin significantly decreased sleep latency, did not exacerbate spike density or seizure frequency, increased the duration of slow-wave sleep and REM latency, and decreased the duration of REM sleep. The authors concluded that a larger scale study of melatonin should consider its safety, tolerability and secondary outcomes in children with epilepsy.

## Adverse effects of melatonin

The most frequently reported side effects associated with the use of melatonin in children include morning drowsiness, increased enuresis, headache, dizziness, diarrhea, rash, and hypothermia [71]; a mild and transient headache and gastrointestinal symptoms mainly occur during the first days of treatment [72].

Melatonin is primarily metabolised by CYP1A2 and CYP2C19, and so inhibitors of CYP1A2 may increase melatonin concentrations [73]. As melatonin may decrease blood pressure or serum glucose levels, particular attention should be paid to patients receiving concomitant treatment with agents that affect these variables [74].

Melatonin has been administered to animals at very high doses of 5–20 mg/kg and even > 100 mg that have proved to have neuroprotective activity without giving rise to any adverse effects. However, this does not mean that the same is true of infants and children. Furthermore, one major problem for both prescribers and patients is the confusing number of over-the-counter preparations available in health food stores whose efficacy varies widely.

## Conclusions

Melatonin can be safe and effective not only in the case of primary sleep disorders, but also for sleep disorders associated with various neurological conditions. However, there is still uncertainty concerning dosing regimens and a lack of other data. The dose of melatonin should therefore be individualised on the basis of multiple factors, including the severity and type of sleep problem and the associated neurological pathology. On the other hand, although the doses of melatonin are normally low, some studies suggested that higher doses of melatonin may be useful mainly in patients with melatonin receptors mutations [29–31]. There is also a need for further studies aimed at identifying the sleep disordered infants and children who will benefit most from melatonin treatment, and determining appropriate doses based on the severity and type of disorder. Furthermore, clinical data in children on melatonin in prolonged-release formulations and on melatonin receptor agonists are needed [75–77].

## Abbreviations

AD: atopic dermatitis
ADHD: attention-deficit/hyperactivity disorder
ASD: autism spectrum disorder
BIC: behavioural insomnia of childhood
GABA: gamma-aminobutyric acid
NDDs: neurodevelopmental disorders
OSA: obstructive sleep apnea
SCN: supra-chiasmatic nucleus
SMS: Smith–Magenis syndrome
